# Novel approaches to estimating abortion incidence

**DOI:** 10.1186/s12978-019-0702-0

**Published:** 2019-04-18

**Authors:** Gilda Sedgh, Sarah C. Keogh

**Affiliations:** Guttmacher Institute, 125 Maiden Lane 7th Floor, New York, NY 10038 USA

**Keywords:** Abortion estimation, Confidante approach, Modified AICM, Studying clandestine behaviors

## Abstract

**Background:**

In countries where abortion is legally restricted or clandestine, estimates of abortion incidence are needed in order to bring attention to the reality of this practice. Innovations in methods for estimating stigmatized behaviors, coupled with changes in the conditions under which women obtain abortions, prompt us to review new approaches to estimating abortion incidence and propose innovations in this field.

**Methods:**

We discuss five approaches for yielding accurate estimates in countries with restrictive abortion laws. These include two prevailing approaches in the field (direct questioning of women about their abortions and the Abortion Incidence Complications Method (AICM)), one that has begun to be in use in recent years (the List Experiment) and two that are newly proposed by the authors (the Confidante Approach and a modification of the AICM). We discuss assumptions, strengths and weaknesses of each approach. Finally, we suggest strategies for assessing the validity of the findings in the absence of a gold standard.

**Results:**

Though direct questioning has consistently been shown to miss many abortions, reporting can potentially be improved by normalizing or reframing the experience of abortion. The AICM has had the advantage of not relying on women’s reports about their abortions; however as self-induced abortion becomes more common, this strength becomes a weakness. The modified AICM, which uses women’s abortion reports to estimate the proportion of abortions that lead to treated complications, improves our chances of capturing self-induced abortions. The List Experiment preserves the woman’s anonymity (not just her confidentiality), but it can be cognitively challenging and the potential to make subgroup estimates is extremely limited. The Confidante Approach entails asking survey respondents about abortions among women who confide in them, rather than their own abortions. An adjustment factor can be applied to estimate the incidence of confidantes’ abortions that are unknown to respondents. This approach relies on the assumption that women know and will report whether their confidantes had an abortion.

In the absence of a gold standard measure of abortion incidence, four strategies can be employed to compare and assess these approaches: (a) comparing the level of underreporting across methods susceptible to underreporting but not to overreporting, (2) validating components of abortion estimates against an objective measure, (3) testing whether these strategies accurately estimate other sensitive behaviors for which a gold standard exists, and 4) sensitivity analyses. Ultimately, it might be appropriate to employ more than one methodology when measuring abortion incidence.

## Plain English summary

In countries where abortion is largely clandestine, it is difficult to estimate its incidence. But such estimates are needed in order to bring attention to this practice. Innovations in methods for estimating stigmatized behaviors, coupled with changes in the conditions under which women obtain abortions, prompt us to review new approaches to studying abortion and to propose innovations in this field.

We review direct questioning of women about their abortions, an indirect approach referred to as the Abortion Incidence Complications Method (AICM), and the List Experiment. We additionally propose the Confidante Approach and a modification of the AICM.

Results from direct questioning could be improved by normalizing or reframing abortions. The AICM uses an estimate of the number of women receiving post-abortion care, taken from a survey of healthcare facilities, multiplied by the inverse of the estimated proportion of abortions that lead to treated complications, taken from a survey of knowledgeable informants. The modified AICM uses women’s abortion reports to estimate the proportion of abortions that lead to treated complications and should improve our chances of capturing self-induced abortions. The List Experiment entails asking women to report, from a list of items, how many items she has experienced but not which ones. It preserves women’s anonymity but is difficult to implement and yields limited information. The Confidante Approach entails asking survey respondents about abortions among their confidantes, rather than their own abortions. An adjustment factor is applied to account for abortions that confidantes do not tell the respondents about. We discuss ways to assess the performance of these approaches, in the absence of a gold standard. Employing more than one methodology when measuring abortion incidence can often be helpful.

## Background

As of 2018, some four in ten women of reproductive age live in the 125 countries with highly restrictive abortion laws [1], and information on abortion incidence is not routinely gathered in such countries. Reliable estimates of abortion incidence are also lacking in many countries where abortion is legally allowed but nevertheless clandestine due to persisting stigma. Yet, estimating the incidence of abortion is a critical step toward bringing attention to the reality of this practice in these settings. Abortion incidence estimates are also a necessary foundation for research on the safety of abortions performed and the consequences of unsafe abortion, and are used in estimating other reproductive health outcomes such as contraceptive failure and unintended pregnancy rates. Moreover, estimates of trends in abortion incidence can contribute to research on the impacts of policies, laws and regulations on abortion.

The clandestine and often illegal nature of induced abortion complicates accurate incidence measurement. One of the most common approaches to measuring abortion incidence in such settings is asking women directly if they have had an abortion. This approach has proven ineffective in most settings because many women are unwilling to admit to having had an abortion [2]. Researchers have attempted to use methods aimed at ensuring the anonymity of respondents, such as the Randomized Response Technique and Sealed Envelope Method (see Appendix), but these have not been demonstrated to consistently generate reliable estimates [3]. Another strategy to reduce respondent bias is the preceding birth technique, which asks women seeking antenatal care whether they had a previous pregnancy and how it ended [4]. However, this does not capture abortions among childless women, or repeat abortions, and therefore does not provide an accurate estimate of incidence.

Other approaches have used model-based methods of estimation. The residual method uses the proximate determinants of fertility model [5] to calculate the abortion rate as the residual contribution to total fertility when the other proximate determinants of fertility are accounted for [6]. However, the abortion rate is very sensitive to inaccurate estimates of the other proximate determinants, which can be a problem in countries with unreliable official statistics, where abortion estimates tend to be most needed.

In the early 1990s, an indirect approach was developed that did not rely on women self-reporting their abortions. In this approach, referred to as the Abortion Incidence Complications Method (AICM), the number of abortions is estimated as the number of women receiving post-abortion care (PAC) in a health care facility (obtained from a survey of health facilities), multiplied by the inverse of the proportion of abortions that lead to facility-based treatment (known as the multiplier, and obtained from a survey of knowledgeable informants) [7]. This approach has been used in more than twenty countries, and modifications and improvements have been made to the method over time.

New methodological approaches to estimating abortion and other clandestine behaviors have been devised and tested in recent years, in particular the List Experiment and network-based approaches such as Anonymous Third Party Reporting (ATPR), the Best Friend Approach and the Network Scale-Up Method (see appendix). These lay the groundwork for further innovation in the field of abortion incidence estimation. Such innovation is all the more critical as the prevalence of self-induced abortions with misoprostol continues to increase, thus drastically changing the context in which abortions occur.

In this paper, we review five methodologies, comprised of two currently prevailing methods, one that has begun being used in recent years, and two new methods that have the potential to yield the most accurate results when implemented in low-resource countries, where these estimates are most needed. Many of the approaches described above are excluded from this review because they have been in existence for several years and previously reviewed [1] and they are already known to be either highly imprecise (model-based approaches), incomplete by design (preceding birth technique), inconsistent in their performance (Sealed Envelope Method) or cognitively challenging in low literacy settings (Randomized Response Technique) (the Network Scale Up Method is also excluded for this reason, though it has not been previously reviewed or widely used in abortion research). The five approaches that met our inclusion criteria were: (1) direct questioning of women about their pregnancy terminations, (2) the AICM, (3) the List Experiment, (4) the Confidante Approach (which builds on the ATPR and the Best Friend Approach), and (4) a modification of the AICM. The first two of these methods are considered the standard in the field and have been employed in many countries; it is therefore important to include them for comparison purposes. The Confidante Approach and modified AICM are newly proposed here. We discuss key assumptions, strengths and weaknesses of the approaches covered in this review. We also suggest strategies for assessing the validity of the findings in the absence of a gold standard.

### Abortion estimation methodologies

#### Direct questioning

Direct questioning involves surveying women and asking them whether they have had an abortion in their lifetime and in a specific period of time, such as in the prior 3 years. Respondents can report multiple abortions and the timing of each one*.* The sampling frame is all women of reproductive age in the country (or other defined geographic area of interest). The sampling strategy should ensure that a representative sample of women in the geographical area is selected. In a community-based survey, this can typically be achieved through a multi-stage sample, first selecting enumeration areas with probability proportional to size, then randomly selecting households within enumeration areas, then selecting women of reproductive age in the household. Data are collected through survey questionnaires that can be interviewer-administered, self-administered through audio computer-assisted technology (ACASI), or by phone. However, interviewer-administered questionnaires are likely to be the most effective in resource-limited countries with low literacy.

While this method is known to underperform as a source of incidence estimates, measures to improve self-reporting include preceding the questions with an introduction that normalizes the experience of unintended pregnancy and abortion. Asking about abortions together with a non-stigmatized behavior is another potential way to normalize abortions to encourage accurate responses. Some Demographic and Health Surveys (DHS) ask whether women have had an abortion or a miscarriage in a single question – but this approach has not been useful for estimating induced abortion rates. Where misoprostol is available, some women might use the drug either with or without a pregnancy test, and some women may view this as a way to initiate the onset of menses rather than as an induced abortion. Thus we propose additionally asking women if they have used medications to bring on their periods, without referencing abortion in this question. Strategies would then be needed for estimating the proportion of such procedures that were actually terminations, using information such as the time since a woman’s last menstrual period, and possibly subsequently asking if she believes she was pregnant at that time.

The annualized abortion rate based on reporting of abortions in the prior 3 years is calculated as:

$$ \left[\#\mathrm{of}\ \mathrm{abortions}\ \mathrm{reported}\ \mathrm{by}\ \mathrm{women}\ \mathrm{in}\ \mathrm{the}\ \mathrm{last}\ 3\ {\mathrm{years}}^{\ast }\ 1000\right]/\left[\mathrm{total}\ \mathrm{women}\ {\mathrm{in}\ \mathrm{survey}}^{\ast }\ 3\right] $$

It is also possible to calculate the lifetime incidence of abortion, and annual rates for each of the past 3 years based on reported timing of abortions. This can help assess whether reporting of abortions decreases with time since the event.

A strength of direct reports is that information can be obtained to allow estimates for population subgroups and for incidence by type of procedure used, such as the proportion of abortions that are induced medically, to the extent that women can accurately report this information. In addition, policy relevant information on the consequences of unsafe abortion and on factors associated with abortion decision-making can be obtained through this method, although this information does not improve incidence estimation.

As already noted, the main weakness of the direct report method is that it has been consistently shown to underestimate the true incidence of abortion [2]. Also, abortion reporting may vary according to women’s characteristics, their abortion experiences and the outcome of their abortion. For example, if more educated women or women who had complications are more likely to report an abortion, then estimates of subgroup rates of abortion or of the proportion of abortions done safely may be biased.

#### The abortion incidence complications method (AICM)

The AICM primarily relies on data from two surveys: a) a nationally representative survey of health facilities, which is used to estimate the number of women who receive PAC in facilities and b) a survey of professionals expected to have some expertise on abortion, which is used to estimate, for each PAC case, how many induced abortions likely occur without complications or with untreated complications. The sampling frame for the health facilities survey is all levels and types of public and private facilities throughout the country (or geographic area of interest) that can be expected to provide PAC and whose catchment area includes the study’s target population. A random sample of facilities is selected, usually stratified by region and type of facility, with separate sampling fractions for each facility type. In each facility, the most qualified staff member is selected to respond to an interviewer-administered structured survey about PAC caseloads. For the survey of knowledgeable informants on abortion, interviewers administer a structured questionnaire to a purposive sample of professionals selected in consultation with in-country stakeholders, sometimes stratified by region. Results from these surveys are combined to compute the number of abortions and abortion rates per 1000 women aged 15–44 for the year preceding the survey.

This methodology provides estimates not only of abortion incidence, but also informants’ assessments of the incidence of treated and untreated complications and of inequities in access to safe abortion and PAC across subgroups of women. A number of assumptions are made to subtract miscarriages from reported facility caseloads and to avoid double counting women who are treated in more than one facility. The method has been adapted to various country contexts in which unsafe abortion is prevalent, including in countries where some women are able to obtain legal abortions in facilities.

A key strength of this method is that the estimate uses information on visible abortions, i.e., PAC caseloads, which can somewhat reliably be obtained through a nationally representative survey of health facilities. The method does not rely on women’s reports about their abortions for any aspect of the estimate, so it may be particularly useful when it is not feasible to conduct a large scale survey of women.

A major limitation of the AICM is that the multiplier is calculated from a purposive sample of informants, and gaps in either sample selection or respondents’ knowledge may lead to biases in the abortion incidence estimation. For example, abortion incidence may be underestimated if many abortions that do not lead to treated complications are done without the knowledge of these informants. Where the incidence of self-induced abortion with misoprostol is widespread, this type of under-estimation is particularly likely to occur. Another limitation of the AICM is that assumptions that are invoked to estimate the proportion of PAC cases that are miscarriages might not be accurate: in the absence of an estimate of the proportion of miscarriages treated in facilities, typically the AICM assumes that the proportion of late miscarriages treated in facilities is similar to the proportion of live births delivered in facilities, and that women do not seek treatment for miscarriages that occur before 13 weeks’ gestation. Also, this method does not provide information on the characteristics of women who have abortions or the contexts in which these abortions occur.

#### List experiment

The List Experiment, also known as the Item Count Technique, has been used to measure the prevalence of various sensitive and stigmatized behaviors and attitudes such as risky sexual behavior [8], drug use [9], and racism [10], and recently abortion incidence in Liberia [11] and Rajasthan [12]. A recently published commentary provides a comprehensive review of considerations relevant to using this approach to study abortion [13]. The List Experiment is conducted as part of a nationally representative community-based survey of women of reproductive age, and involves reading to respondents a list of three or four non-sensitive events (for example staying overnight in hospital) and asking them *how many* of these events they have experienced, but not which ones. In half of the sample (the treatment group), abortion is added as an additional item on the list. The incidence of abortion is calculated as the difference between the mean number of events experienced by the treatment group and the control group. To increase the power of the estimate, investigators have used the “double List Experiment,” [11, 14] whereby two different lists (A and B) are included in the questionnaire, and each group is a treatment group for one list and a control group for the other list. The overall incidence of abortion is then calculated as the mean of the estimates derived from each list.

Strengths of this approach include that it obtains information directly from women without asking them to disclose to the interviewer whether they had an abortion. The estimate from the List Experiment should be less prone to underreporting than direct questioning if women trust that their anonymity will be preserved. This approach is more appropriate for illiterate women than the Randomized Response Technique, and it might be more likely to elicit trust than the Sealed Envelope Method. It can be used to estimate sub-regional rates as well as subgroup abortion rates for large subgroups.

Limitations include that it cannot capture multiple abortions obtained by the same woman, and it does not provide information on the characteristics of women who have had abortions. Moreover, though the approach can be used to ask whether a woman had an abortion in a specified window of time, it does not lend itself to additional questioning strategies to improve women’s recollection of when the abortion took place. It can also be cognitively challenging [15]. Moreover, women may still not trust that their responses are anonymous, and therefore might not disclose their abortions. Previous studies have not found a consistent relationship between the List Experiment estimates and direct reports: in Liberia the abortion rate estimated from the List Experiment was five times higher than the rate estimated from direct self-reports, while in Rajasthan the List Experiment led to a lower estimated rate than self-reports [11]. Testing of this approach is currently underway in Ghana.

#### Confidante approach

This approach entails asking women about the abortions in their social networks, rather than their own abortions, as with direct questioning. Data are collected through a nationally representative, community-based, interviewer-administered survey of women of reproductive age, with the sampling frame being all women of reproductive age in the geographic area of interest. Each respondent is asked to think of up to three women aged 15–49 who live in the area of interest and who would share their secrets with her, and with whom she would also share her secrets. For each confidante, information is collected on her sociodemographic characteristics and whether she has had an abortion in a designated window of time. This approach combines the strengths of the Best Friend Approach [16] and the Anonymous Third Party Reporting (ATPR) approach [12], both of which have been used previously in abortion research; the former only asks the respondent about a single best friend (who may or may not be a confidante), and the latter asks the respondent about abortions among all her confidantes (see Appendix).

Many respondents will not know whether their confidantes had an abortion; these confidantes will be removed from both the denominator and the numerator of the estimated rate. This will affect the precision, but not the validity of the estimate of abortion incidence. Respondents are also asked whether they are certain about their confidante’s abortion or only suspect it occurred. Some respondents will say that their confidante did not have an abortion when in fact they do not know that their confidante had an abortion. Drawing from the network-scale up method, we can apply a visibility factor to correct for bias resulting from this misreporting [17]. This factor can be computed by asking respondents who have had an abortion whether they told their confidante about their abortion; the visibility factor is the inverse of the proportion of respondents who told their confidantes about their abortion. For example, if 50% of respondents who had an abortion told their confidantes about the abortion, we would assume that 50% of confidantes told the respondents about their abortions, and we would multiply the abortion rate among confidantes by (1/0.5), or two. A separate visibility factor can be computed for the first, second and third confidante. Thus, if there is attrition in knowledge and reporting of abortions with each successive confidante, we would compute and apply a larger visibility factor for the group of “third confidantes,” and the findings from this pool of confidantes would still be useful.

Asking about more than one confidante can increase the sample size relative to the Best Friend Approach and self-reports. By limiting the confidantes to a fixed number, we avert the key limitation of the ATPR, which relies on the respondent accurately listing all of her confidantes who would confide their abortions to her. The findings from the ATPR in Burkina Faso indicated that women have 1.7 confidantes on average [18]. If confidantes are not representative of the general population of women of reproductive age – for example, if confidantes are younger or older than the general population on average – the sample should be weighted in order to better match the distribution of respondents.

The annualized abortion rate in the 3 years before the survey is computed as:

$$ \left[\#\mathrm{of}\ \mathrm{friends}\ \mathrm{who}\ \mathrm{had}\ \mathrm{an}\ \mathrm{abortion}\ \mathrm{in}\ \mathrm{previous}\ 3\;{\mathrm{years}}^{\ast }1000\right]/\left[\mathrm{total}\#{\mathrm{friends}}^{\ast }3\right] $$

As with direct reporting, the annual abortion rate can also be calculated separately for each of the three preceding years based on reported timing of the confidante’s abortion(s), although these annual rates will have lower power.

This approach relies on the assumptions that (a) the probability that a woman will name a person as her confidante is independent of the probability that the confidante has had an abortion, and (b) respondents are willing to report the abortions they know that their confidantes had. To help ensure that a woman’s probability of being named as a confidante is independent of the probability that she has had an abortion, it is important to phrase the initial confidante-generating question in such a way that the respondent does not suspect the interviewer will be asking about abortions specifically. It is also useful to place the questions about confidantes before any other direct questions about abortion in the questionnaire. Furthermore, it is best if the introduction to the survey does not convey that abortion is the primary the focus of the research endeavor.

To examine whether the respondents know about the hidden reproductive behaviors of their confidantes, interviewers can ask respondents about their confidantes’ contraceptive use and compare the contraceptive prevalence among confidantes to the population prevalence from the DHS; this comparison can further be made for sociodemographic subgroups of women.

Women might selectively disclose their abortions to others who are least likely to disapprove of the abortion [19]. We can examine whether this is the case by asking women about their attitudes towards abortion, and comparing abortion rates among confidantes of respondents who have negative versus permissive attitudes toward abortion.

Strengths of the confidante method are that it is easier to achieve statistical power with this method relative to direct reporting, and it allows for an analysis of the basic characteristics of women who have abortions. In addition, respondents can report multiple abortions for each confidante. It also potentially reduces the effect that stigma plays on abortion reporting. As with direct reports of abortion, the confidante method allows for a more accurate estimation of the timing of the abortion than the List Experiment. The confidante method can also collect information on the proportion of confidantes’ abortions that had complications and received PAC. This proportion can be used as the multiplier for the modified AICM instead of, or in addition to, respondents’ reports on their own abortions – if respondents are not disproportionately more likely to know about the complicated abortions that their confidantes had.

One limitation of the Confidante Approach is that it yields limited information on the abortion process, such as the provider and method used. Also, it is possible that abortions that lead to complications are more likely to be visible to and known by the respondent, thus estimates of the proportion of abortions that are done safely based on the Confidante Approach might be biased.

#### Modified AICM

To address some of the limitations in the AICM, we propose a modified version of the AICM. As with the traditional AICM, estimated PAC caseloads would be gathered from a nationally representative sample of health facilities. However, the number of abortions not receiving PAC for every abortion receiving such care would be obtained from women’s self-reported abortions instead of from knowledgeable informants. Women’s self-reports would be collected through a nationally representative community-based survey as described in the section on direct reporting. Another approach that has not been tested is asking respondents how many abortions have taken place in their community, and how many of those have led to a treated complication. Though complicated abortions are more likely to be known to respondents, the magnitude of the resulting bias might be lower than it is in the survey of knowledgeable informants. The sampling frame is all women of reproductive age in the country.

A survey of women can also yield an estimate of miscarriage treatment rates if women are asked whether they have had a miscarriage and whether they sought treatment for it at a health care facility. However, the fact that DHS estimates of miscarriage incidence tend to be higher than the expected population incidence suggests that some women classify their abortions as miscarriages when responding to the survey questions. It would therefore be important to devise a questioning approach that will minimize this bias. For example, when asking about miscarriages, interviewers can specifically ask women to not include any induced abortions in their responses.

The modified AICM assumes that women’s reporting of their abortions is independent of whether they ultimately received PAC. This assumption may not hold, for example, if women are less likely to report uncomplicated abortions than complicated abortions; however, tabulations of data from surveys of women in Burkina Faso [18] and Nigeria [20] suggested there is less bias from this source than from the survey of knowledgeable informants.

Table 1 presents the methods reviewed here, along with indications of the expected direction and magnitude of bias associated with each method, the expected precision, and other key points about the methods.

**Table 1:** Comparison of abortion incidence estimation methodologies

	Expected direction of bias	Expected magnitude of bias	Relative precision	Ability to capture self-induced abortions known only to woman	Potential to yield contextual information	Key data sources
**Methods reviewed in this paper**						
Abortion incidence complications method	Unknown	Unknown	Moderate	Only if led to PAC	From PAC facilities and knowledgeable informants	Health facility survey, survey of knowledgeable informants
Direct questioning	Underestimation	Very high	Very low	Yes	From women who admit to having an abortion	Population-based survey of women
List experiment	Underestimation	Low	Low	Yes	No	Population-based survey of women
Confidante method	Underestimation	Moderate	High	No	Limited	Population-based survey of women
CM with visibility factor	Unknown	Low	High	Yes	Limited	Population-based survey of women
Modified AICM	Unknown	Unknown	Low /moderate	Yes	From PAC facilities and women who admit their abortions	Health facility survey, population-based survey of women
**Other methods**						
Preceding birth technique	Underestimation	High	Low	Yes	From women who admit to having an abortion	Suvey of antenational clinic patients
Sealed envelope	Underestimation	Inconsistent	Low	Yes	No	Population-based survey of women
Randomized response technique	Unknown	Unknown	Low	Yes	No	Population-based survey of women
Residual method	Unknown	Unknown	Very low	Yes	No	Secondary data
Best friend approach	Underestimation	Low/moderate	Moderate	No	Limited	Population-based survey of women
Sealed envelope	Underestimation	Moderate	Very high	No	Limited	Population-based survey of women
Network scale-up	Unknown	Unknown	Very high	Yes	No	Population-based survey of women

### Determining the best approaches for estimating abortion incidence

In the absence of a gold standard measure of abortion incidence, four general strategies described below can be employed to assess and compare how the approaches perform.

#### Comparing the level of underreporting across methods

If selection bias did not result in the women or confidantes who have had an abortion being more likely to be part of the study, the main potential source of bias in the List Experiment, Confidante Method (before adjusting with a visibility factor) and direct questioning is underreporting. A prior review of surveys showed that abortion has consistently been underreported in surveys of women, across a variety of settings [2]. As has been done previously when comparing methodologies for measuring a sensitive practice prone to underreporting [21], it might be reasonable to deem the highest estimated abortion rate from these approaches to be the least biased estimate. We can also compare estimates for demographic subgroups, to assess if the methods rank differently for different subgroups.

A key concern with respect to the AICM is that experts will not be fully aware of the prevalence of abortions that do not result in treated complications (including abortions using misoprostol). In the modified AICM women are also expected to underreport self-induced abortions more than they underreport abortions that led to complications, if they have succeeded thus far in keeping uncomplicated abortions a secret. Thus the approach that yields the higher multiplier – or the higher number of untreated abortions for every abortion that leads to PAC in a facility – could be deemed the more valid estimate, at least with respect to the multiplier. Since informants are asked to estimate uncomplicated abortions for different socio-demographic subgroups of women, we can also compare informants’ estimates to those from self-reports for these different subgroups.

#### Validating components of abortion estimates against an objective measure

With data from the HFS as a gold standard for the measure of the incidence of abortions that lead to facility-based PAC in the country, estimates of PAC treatment rates from the HFS can be compared with estimates based on the confidante method and self-reporting. The estimate based on the HFS includes miscarriages; reports of treated miscarriages can be added to the estimates derived from the other two sources, or treated miscarriages can be subtracted from the HFS, though different estimates of miscarriage treatment rates are possible.

In order to ascertain the validity of miscarriage estimates taken from direct reports, we can compare them with the population incidence of miscarriage estimated from life tables [5]. Life tables are not necessarily a gold standard, but they are to-date the best estimates available of the incidence of miscarriage.

#### Testing the validity of estimates of other sensitive reproductive health behaviors

To test the Confidante Approach and List Experiment, they can be used to estimate the incidence or prevalence of other sensitive behaviors for which the true incidence is known. They can be used, for example, to ask about contraceptive prevalence, and results can be compared with contraceptive prevalence estimates from the DHS.

#### Sensitivity analyses

While these are not part of the validation process, sensitivity analyses can help determine the influence of certain components of the calculations on the overall incidence estimates. For example, investigators can compare how the results of the AICM and modified AICM vary according to the source of miscarriage estimates, and whether the estimate from the List Experiment is sensitive to the nature of the non-sensitive questions asked alongside the one on abortion.

## Discussion

Investigators have made much headway in studying abortion in restrictive settings, and there is yet more progress to be made in this field of study. Findings gleaned from these efforts can potentially also inform efforts to study other clandestine or stigmatized behaviors.

In assessing these estimation methods, a central criterion is the validity of the resulting measure of abortion incidence. But other factors merit consideration in determining the best way to use limited resources to study abortion. These include a consideration of whether the research method allows investigators to accurately classify when abortions occurred; whether the methodology can be employed across a range of settings (and thus whether they allow for comparison of estimates across settings); the level of anonymity retained for women who have abortions; the cost required to achieve comparable levels of precision across methods; the ability of the study design to yield contextual information on abortion which is also of policy relevance; and whether the approach can reasonably be used to assess trends in abortion incidence, even in the face of some bias in point estimates.

We excluded from this review methods that are cognitively challenging and seem unlikely to work in low resource settings. It is worth noting that the network scale-up approach is currently being tested to study abortion incidence in Ethiopia and Uganda [23]. If the findings are promising, further consideration of the approach will be warranted.

Ultimately, it might be appropriate to employ more than one methodology whenever undertaking a study of abortion incidence. For example, the Confidante Method can serve as a warm up to questions about respondents’ own abortion experiences and thus help improve the quality of self-reports, and self-reporting is a required component of the modified AICM. Self-reporting can also represent a source of contextual information on abortion, in a study that employs another method to estimate abortion incidence. Research using multiple methods is underway in Ghana, Indonesia, Uganda, Ethiopia, Nigeria, Cote d’Ivoire and Rajasthan (India). The findings from those studies provide opportunities to compare how the approaches fare, and could help us glean additional insights into these estimation techniques.

In the years ahead, the stigma associated with abortion might be lifted, laws might become less restrictive in many countries, and obtaining an abortion might become a relatively simple process. It is alternatively possible that the proportion of abortions that are unsafe, clandestine and illegal will increase, if laws, policies and social mores become more conservative over time. However the landscape of abortion provision evolves, accurate estimates of abortion will be key to informing policy and programs. As new research methods continue to emerge, it will be important for researchers to continue to challenge themselves to measure abortion using the most rigorous approaches available.

## APPENDIX

### Randomized Response Technique

In the Randomized Response Technique, the survey respondent randomly and secretly selects which of two questions she will respond to: a non-sensitive question with a known response probability and a question about whether she has had an abortion. Key limitations are that a very large sample size is required, since some women will respond only to the non-sensitive question, and it requires the respondent’s understanding of the instructions and trust in the approach. In addition, it is not possible to estimate subgroup or sub-regional abortion rates. Comparisons indicate that the approach identifies more abortions than does direct questioning [4, 24], but that incidence estimates can be unrealistically low [4].

### Sealed Envelope Method

The Sealed Envelope Method entails asking women to respond to questions about abortion in a very short, self-administered instrument and return it in an unmarked sealed envelope. This method is difficult to administer to illiterate women, and its effectiveness depends on the respondent’s trust in the anonymity of her responses. It identified more abortions than direct questioning, but far fewer than the AICM, in the Philippines [25] and Nigeria [26], and it elicited even fewer abortions than direct reporting in Zambia [26].

### Anonymous Third Party Reporting (ATPR)

The ATPR entails asking women in a survey to think about all their confidantes, and then asking questions about each confidante, including whether she had an abortion [12, 18, 27]. The abortion rate in the population is computed as the rate among all the confidantes. The ATPR has relatively strong statistical power, because each respondent provides information on multiple women. A key limitation is that the denominator of the computed abortion rate is sensitive to whether the respondent gives an accurate listing of women who would tell her if they had an abortion (for example, women who had an abortion might be overrepresented if the fact of sharing her abortion experience prompts the respondent to name her as a confidante). The ATPR has been employed in Ouagadougou, where it generated a very high incidence estimate [27], nationally in Burkina Faso, where it yielded an estimate slightly lower than the AICM [18], and in Rajasthan, India where the resulting estimate was lower than that from self-reporting [12]. This variability could stem from differences in the network generating question, or differences in the extent to which women tell others about their abortions.

### Best Friend Approach

In this approach, each woman in a community-based survey is asked to think about the woman who is her closest friend (or relative) of reproductive age, and whether that friend has had an abortion [16]. The abortion rate is computed as the rate among the best friends. This method has limited power, since each questionnaire yields information about only one woman. It also assumes that the women whom respondents list as their best friend are a representative sample of reproductive age women, which may not be true if some women are listed more than others and these women have different abortion experiences than the general population of women. This approach was employed in Malawi [16], where it yielded higher estimates of abortion than self-reports.

### The network scale-up method (NSUM)

This approach entails asking the respondent to count all the women in her social network who have had an abortion in a particular window of time [28]. Her social network might be defined, for example, as all women who the respondent has had a meal with in the past year. The respondent’s total network size relative to the size of the population is calculated either by estimating how many people she knows in a population with a known size (e.g. women named Grace) averaged out over many different known-size populations, or by asking how many people the respondent knows in many small, mutually exclusive subgroups (e.g. family members, non-family neighbors). The NSUM assumes that (a) respondents’ networks are accurately reported on average, (b) the networks are representative of the general population in which the respondents live, (c) women who had abortions have the same network size as the general population, and (d) respondents would know if their connections had had an abortion. Adjustments have been proposed to correct for the last two of these potential sources of bias [22]. A long list of questions is needed in order to ascertain a woman’s network size and adjust for potential biases.

## Abbreviations

AICM: Abortion Incidence Complications Method
ATPR: Anonymous Third Party Reporting
PAC: Post abortion care
